# Evaluation of pathotype marker genes in *Streptococcus suis* isolated from human and clinically healthy swine in Thailand

**DOI:** 10.1186/s12866-023-02888-9

**Published:** 2023-05-16

**Authors:** Anusak Kerdsin, Nichari Bamphensin, Kulsatri Sittichottumrong, Ratchadaporn Ungcharoen, Parichart Boueroy, Peechanika Chopjitt, Rujirat Hatrongjit, Marcelo Gottschalk, Nuchsupha Sunthamala

**Affiliations:** 1 Faculty of Public Health, Kasetsart University Chalermphrakiat Sakon Nakhon Province Campus, Sakon Nakhon, 47000 Thailand; 2 Faculty of Science and Engineering, Kasetsart University Chalermphrakiat Sakon Nakhon, Province Campus, Sakon Nakhon, 47000 Thailand; 3 Groupe de recherche sur les maladies infectieuses en production animale (GREMIP), The Swine and Poultry Infectious Diseases Research Centre (CRIPA), Faculty of Veterinary Medicine, University of Montreal, Québec, Canada; 4 Department of Biology, Faculty of Science, Mahasarakham University, Mahasarakham, 44150 Thailand

**Keywords:** *Streptococcus suis*, Serotype, Sequence type, Pathotype, Multiplex PCR

## Abstract

**Background:**

*Streptococcus suis* is a zoonotic pathogen that causes substantial economic losses in the pig industry and contributes to human infections worldwide, especially in Southeast Asia. Recently, a multiplex polymerase chain reaction (PCR) process was developed to distinguish disease-associated and non-disease-associated pathotypes of *S. suis* European strains. Herein, we evaluated the ability of this multiplex PCR approach to distinguish pathotypes of *S. suis* in Thailand.

**Results:**

This study was conducted on 278 human *S. suis* isolates and 173 clinically healthy pig *S. suis* isolates. PCR identified 99.3% of disease-associated strains in the human isolates and 11.6% of non-disease-associated strains in the clinically healthy pig isolates. Of the clinically healthy pig *S. suis* isolates, 71.1% were classified as disease-associated. We also detected undetermined pathotype forms in humans (0.7%) and pigs (17.3%). The PCR assay classified the disease-associated isolates into four types. Statistical analysis revealed that human *S. suis* clonal complex (CC) 1 isolates were significantly associated with the disease-associated type I, whereas CC104 and CC25 were significantly associated with the disease-associated type IV.

**Conclusion:**

Multiplex PCR cannot differentiate non-disease-associated from disease-associated isolates in Thai clinically healthy pig *S. suis* strains, although the method works well for human *S. suis* strains. This assay should be applied to pig *S. suis* strains with caution. It is highly important that multiplex PCR be validated using more diverse *S. suis* strains from different geographic areas and origins of isolation.

**Supplementary Information:**

The online version contains supplementary material available at 10.1186/s12866-023-02888-9.

## Introduction

*Streptococcus suis*, a zoonotic gram-positive coccus bacterial pathogen, causes significant economic losses in the pig industry and invasive infections in humans who are in close contact with infected pigs or contaminated pork-derived products [1, 2]. Among the 29 true serotypes, serotypes 2 and 14 are the most pathogenic in humans [1, 2], and the serotypes most commonly isolated from diseased pigs are serotypes 1/2, 2, 3, 7 and 9 [1].

The identification and characterization of virulence factors or markers in *S. suis* would be a major advance in understanding the pathogenesis of infection and would aid in epidemiological surveillance. There is still no consensus on universal factors or markers that can clearly differentiate pathogenic from nonpathogenic isolates. Molecular tools to differentiate potentially pathogenic pathotype (disease-associated) strains from nonpathogenic pathotype (non-disease-associated) strains are of utmost importance [3]. Currently, there are several methods that claim to determine or predict pathogenic *S. suis* strains, including genotyping of *epf, mrp*, and *sly* [4, 5]; MLST [6]; whole-genome sequencing [7–9]; minimum core genome (MCG) [10]; serotyping PCR [11, 12]; five newly proposed virulence-associated genes (VAGs), including *srtF*, *ofs*, RNA-binding protein (*SSU_RS09525*), and two hypothetical proteins (*SSU_RS09155* and *SSU_RS03100*) [13, 14]; and pathotyping PCR [15]. Some techniques are difficult to apply to the routine testing of a large number of isolates, have high costs and are labour intensive and time-consuming. Some techniques lack the discriminatory power to differentiate *S. suis* strains into virulent and avirulent subpopulations, thus limiting their usefulness in epidemiological studies.

Wileman et al. (2019) developed multiplex PCR pathotyping to target three genetic marker genes associated with observed clinical phenotypes, including the genes for copper-exporting ATPase 1, a type I restriction-modification system S protein, and a putative sugar ABC transporter [15]. That study used the selected genetic markers to differentiate *S. suis* into a disease-associated group (pathogenic pathotype/virulent) and a non-disease-associated group (nonpathogenic pathotype/nonvirulent) [15]. Multiplex PCR pathotyping worked well for *S. suis* strains from England and Wales [15], but contradictory results were observed with *S. suis* strains from Switzerland [16]. In addition, this multiplex PCR assay was not validated in *S. suis* strains from geographic regions other than Europe. This study was to evaluate the ability of the multiplex PCR pathotyping approach described by Wileman and colleagues [15] to distinguish pathotypes of *S. suis* isolates from Thailand. We used this method with *S. suis* isolates recovered from humans and clinically healthy pigs in Thailand. The current study aimed to determine whether PCR can predict the potential virulence of Thai isolates and provide evidence of the versatility of the multiplex PCR scheme in Thai *S. suis* strains in contrast to European strains.

## Materials and methods

### Bacterial strains and DNA extraction

The *S. suis* strains used in this study were collected from human patients and clinically healthy pigs. Human *S. suis* strains were randomly selected from a previous study [17] that collected strains between 2009 and 2012 in all parts of Thailand. Healthy pig *S. suis* strains were collected between April 2010 and March 2011 in northern Thailand [18]. The criteria for selection were based on the distribution of serotypes, sequence types (ST), clonal complexes (CC), area (provinces/regions), isolation years, and sources.

In total, 451 *S. suis* strains were selected and used in this study, consisting of human isolates (n = 278) and clinically healthy pig isolates (n = 173). Among these, 239 were serotype 2 isolates (human = 226; pig = 13), 47 were serotype 14 isolates (human = 46; pig = 1), 14 were serotype 9 isolates (human = 1; pig = 13), 7 were serotype 4 isolates (human = 1; pig = 6), 6 were serotype 7 isolates (pig only), 4 were serotype 5 isolates (human = 2; pig = 2), 4 were serotype 1 isolates (human = 2; pig = 2), 31 were serotype 16 (pig only), 19 were serotype 3 (pig only), 19 were serotype 31 (pig only), and 61 were other serotype isolates from pigs. Details of these isolates are shown in Tables 1 and 2.

**Table 1:** Distribution of pathogenic pathotypes in *Streptococcus suis* isolates from humans

Serotype	CC	ST	Multiplex PCR		No. of isolates	Total
			Type of pathotype	PCR product size (bp)		
1	1	105	I	211, 347	2	2
2	1	1	I	211, 347	32	62
			II	190, 347	2	
			III	211	3	
			IV	190	25	
		126	I	211, 347	2	2
		144	I	211, 347	5	5
		298	I	211, 347	1	1
		337	I	211, 347	5	5
	25	25	IV	190	18	22
			III	211	4	
		103	IV	190	4	6
			III	211	2	
		380	IV	190	7	8
			I	211, 347	1	
		381	IV	190	4	4
		395	IV	190	1	1
		515	IV	190	1	1
		516	IV	190	1	1
	28	28	IV	190	7	8
			II	190, 347	1	
		382	IV	190	2	2
	104	104	IV	190	40	60
			III	211	11	
			I	211, 347	6	
			II	190, 347	3	
		391	IV	190	7	7
		392	IV	190	4	4
		393	IV	190	1	1
		512	IV	190	6	6
		513	IV	190	1	1
		514	IV	190	1	1
	233/379	233	IV	190	11	13
			II	190, 347	2	
		233	Undetermined	211, 892	1	1
		379	IV	190	4	4
4	94	94	II	190, 347	1	1
5	221/234	221	III	211	1	1
	-	235	Undetermined	190, 892	1	1
9	16	16	III	211	1	1
14	1	11	I	211, 347	2	2
		105	I	211, 347	43	43
		237	I	211, 347	1	1
			**Total**		**278**	

**Table 2:** Distribution of pathogenic pathotypes in *Streptococcus suis* isolates from pigs

Serotype	Multiplex PCR		No. of isolates	Total
	Type of pathotype	PCR product size (bp)		
1/2	IV	190	1	1
1	I	211, 347	1	2
	IV	190	1	
2	I	211, 347	4	13
	II	190, 347	1	
	III	211	3	
	IV	190	4	
	Non-disease-associated	892	1	
3	III	211	5	19
	IV	190	13	
	Undetermined	211, 892	1	
4	II	190, 347	1	6
	III	211	1	
	IV	190	4	
5	IV	190	1	2
	Undetermined	190, 892	1	
7	III	211	1	6
	IV	190	3	
	Undetermined	211, 892	1	
	Non-disease-associated	892	1	
8	IV	190	1	7
	Undetermined	190, 892	3	
	Non-disease-associated	892	3	
9	III	211	5	13
	IV	190	3	
	Undetermined	211, 892	2	
	Non-disease-associated	892	3	
11	Undetermined	190, 892	2	5
	Non-disease-associated	892	3	
14	I	211, 347	1	1
15	III	211	2	8
	IV	190	1	
	Undetermined	190 or 211, 892	3	
	Non-disease-associated	892	2	
16	III	211	11	31
	IV	190	15	
	Undetermined	190, 892	5	
17	III	211	2	2
18	III	211	4	5
	IV	190	1	
19	III	211	3	4
	IV	190	1	
24	IV	190	3	3
28	Undetermined	190, 892	2	3
	Non-disease-associated	892	1	
29	III	211	2	9
	IV	190	4	
	Undetermined	190 or 211, 892	3	
31	I	211, 347	2	19
	III	211	3	
	IV	190	5	
	Undetermined	190 or 211, 892	7	
	Non-disease-associated	892	2	
Nontypable	III	211	9	14
	IV	190	1	
	Non-disease-associated	892	4	
	***Total***			***173***

All *S. suis* strains were cultured on sheep blood agar at 37 °C for 24 h. *S. suis* DNA was extracted using the ZymoBIOMICS DNA Miniprep Kit (ZymoBIOMICS DNA Miniprep Kit; Irvine, CA, USA) following the manufacturer’s instructions. *S. suis* species and serotypes of the extracted DNA were confirmed by PCR [11].

### Pathotyping PCR

The multiplex PCR procedure was slightly modified from the previously described procedure [15]. The reaction mixture (15 µl) contained 1X PCRBIO HS Taq Mix Red master mix (PCRBIO Taq DNA Polymerase; London, UK), 0.7 µM of primers SSU1589_0460F and SSU1589_0806R, 0.3 µM of other primers, and 15 ng of template DNA. The primers for pathotyping (pathogenic pathotype [disease-associated] and nonpathogenic pathotype [non-disease-associated]) and their sequences were described in a previous report [15]. The primers included SSU0207_0735F (5’-TTACAAGAACAGGGCAAGACAGTCGCC-3’), SSU0207_0945R (5’-GCTGCTTTATAATCTGGGTGTTCGTTG-3’), SSU1589_0460F (5’- CCTTTAATGCAGGGGACAAAAGTGAGCTC-3’), SSU1589_0806R (5’- CCCATAATCTTACAGTTAACTTCCTTGC-3’), SSUST30534_0368F (5’- ATCCCCTCCCAATAAAAGATTTGGATGC-3’), SSUST30534_1259R (5’- TTTTCGAGCTCTCCATACACTGCTTCTG-3’), SSU0577_0086F (5’- CAGGTAGTTTGGGCTTAGCTTCATCAGG-3’), and SSU0577_0807R (5’- TGGATGCTGAATTCGCAACTGGGCAATC-3’).

The PCR amplification conditions were described previously [15] and consisted of 95 °C for 5 min; 35 cycles of 95 °C for 30 s (denaturation), 66 °C for 90 s (annealing), and 72 °C for 90 s (extension); and a final extension of 68 °C for 10 min. The PCR products were evaluated in 2% agarose gels at 100 V for 30 min. *S. suis* strain P1/7 was used as a positive control for the multiplex PCR. A negative control consisting of the same reaction mixture but with water instead of template DNA was included in each run. The gel was stained with ethidium bromide for 20 min. DNA bands were visualized and photographed under ultraviolet light using gel documentation equipment (SynGene; Cambridge, UK). The sizes of the PCR products were determined by comparison with a molecular size standard (GeneRuler 100 bp Plus DNA ladder; Thermo Fisher Scientific, CA, USA).

**Fig. 1:**
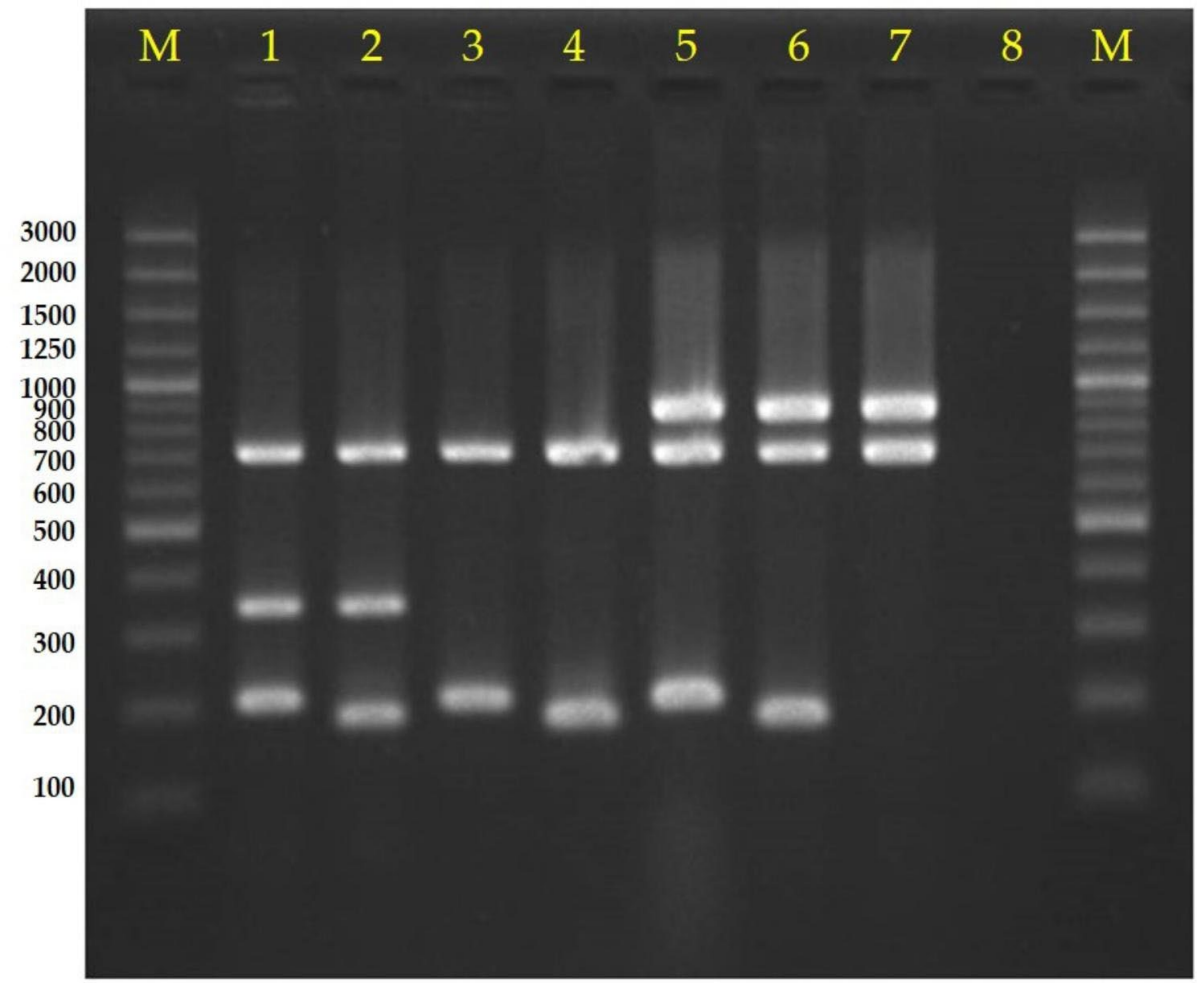
Agarose gel electrophoresis of representative *Streptococcus suis* pathotypes. Lane 1 = disease-associated group I, Lane 2 = disease-associated group II, Lane 3 = disease-associated group III, Lane 4 = disease-associated group IV, Lanes 5 and 6 = undetermined, Lane 7 = non-disease-associated group, Lane 8 = negative control, and Lane M = 100 bp DNA ladder

As shown in Fig. 1, PCR product sizes of both 347 bp and 211 (or 190 bp) or single products of either 347 bp or 211 bp (or 190 bp) were indicative of the disease-associated group (SSU0207: copper exporting ATPase 1 and SSU1589: type I restriction-modification system S protein), while an 892 bp product corresponded with the non-disease-associated group (SSUST30534: putative sugar ABC transporter) [15]. Amplicons of 722 bp corresponded to the *S. suis* species-specific marker (SSU0577: WhiA sporulation regulator) [15]. Diagnostic accuracy was measured in terms of sensitivity, specificity, positive predictive value, and negative predictive value compared to the origin of isolates from either human patients or clinically healthy pigs. Statistics were analysed by Fisher’s exact test with Stata version 17.0 software (StataCorp, College Station, TX, USA). Data were considered significant at *p* < 0.01.

## Results and discussion

This is the first study to utilize PCR pathotyping to test *S. suis* isolates from a region (Thailand) other than Europe (England, Wales, and Switzerland) [15, 16]. Collectively, the current study revealed that the Wileman multiplex PCR pathotyping assay could distinguish all 451 *S. suis* strains into 4 types of disease-associated groups: type I (211 and 347 bp), type II (190 and 347 bp), type III (211 bp), and type IV (190 bp), as shown in Fig. 1. In the case of a PCR product size of either 190 or 211 bp, 892 bp was considered undetermined in this study (Fig. 1). Types III and IV revealed only a single band of either 211 or 190 bp, with no amplification of 347 bp. Two previous studies have also detected 211 or 190 bp bands without a 347 bp band [15, 16].

**Fig. 2:**
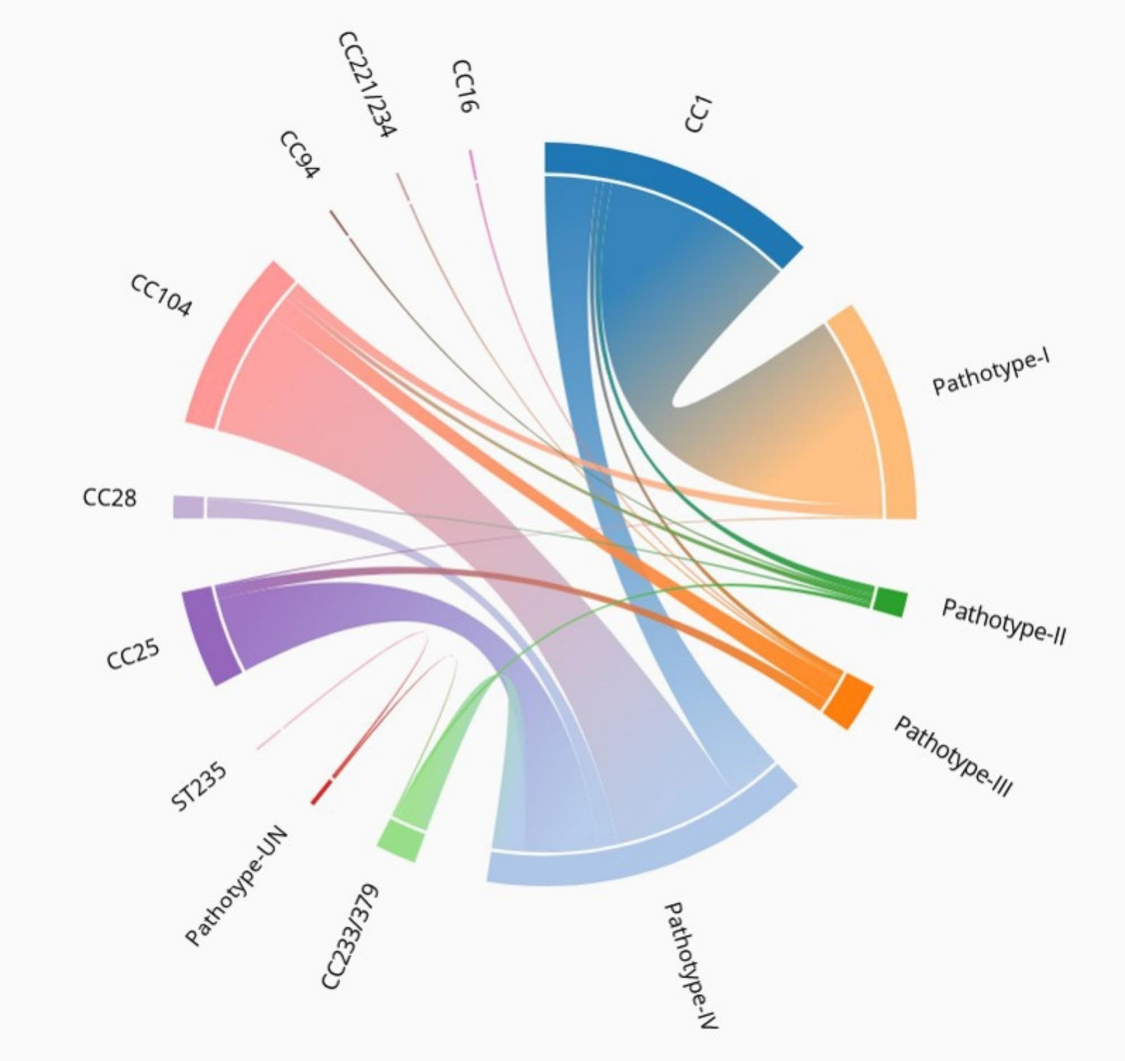
A chord diagram showing the interrelationship between clonal complexes (CCs) and the pathotype of human *S. suis* strains in the current study

The human *S. suis* isolates (n = 278) contained 226 serotype 2 isolates, 46 serotype 14 isolates, 2 serotype 1 isolates, 2 serotype 5 isolates, and one isolate each of serotypes 4 and 9. Of these, 99.3% (n = 276) belonged to the disease-associated group, including 52.6% type IV (n = 145), which was most commonly found in the serotype 2-ST104 strain (n = 40); 36.2% type I (n = 100), which was most commonly found in the serotype 14-ST105 strain (n = 43); 7.9% type III (n = 22), which was found in the serotype 2-ST104 strain (n = 11); and 3.3% type II (n = 9) (Table 1; Fig. 2). We also detected 0.7% (n = 2) that seemed to be undetermined, as shown in Table 1. None of the human isolates were in the non-disease-associated group. This confirmed the PCR results that these groups of isolates were pathogenic and had potential virulence. Hence, the diagnostic sensitivity was 99.3% (276/278), and the positive predictive value was 100% (276/276), whereas the specificity and negative predictive value could not be calculated because all tested *S. suis* isolates were from human patients and were considered pathogenic or virulent. A limitation of this study is that almost 98% of human isolates belonged to either serotype 2 or 14, which represents a relatively homogenous population. It is unknown if this test can be successfully used with isolates recovered from diseased pigs and those of different serotypes. Indeed, Scherrer et al. (2020) demonstrated that the multiplex PCR pathotyping method may work for serotypes 2 and 9 but not for serotypes 1, 3, 6, 7, 15 and 16 [16].

Furthermore, statistical analysis revealed that human *S. suis* CC1 isolates were significantly more likely to be type I disease-associated group rather than any other type. Conversely, CC104 and CC25 were significantly more likely to be type IV than any other type (Table 3). A previous study identified a gene variant of copper ATPase 1 in the pig *S. suis* isolates CC28, CC1109, and serotype 9-ST1105 [16]. In contrast, in the current study, the human serotype 2 isolates ST1, CC25, CC28, CC104, and CC233/379 were more likely to contain the copper ATPase 1 gene variant (Type II or IV) than other ST or CC.

**Table 3:** Relationship of pathotypes and clonal complexes of human *Streptococcus suis* isolates

Clonal complex	N	Type of pathotype			*p* value
		**I**	**IV**	**Other**	
CC1	123	93	25	5	< 0.001^a^
CC104	80	6	60	14	< 0.001^b^
CC25	42	1	35	6	< 0.001^c^
CC233/379	18		15	3	1.00
CC28	11		10	1	1.00
CC221/234	2			2	-
CC16	1			1	-
CC94	1			1	-
	**278**				

^a^ significant association with type I

^b^ significant association with type IV

^c^ significant association with type IV

Surprisingly, when studying the clinically healthy pig isolates (n = 173), 71.1% (n = 123) were classified in the disease-associated group, consisting of 50.4% type IV (n = 62), followed by 41.5% type III (n = 51), 6.5% type I (n = 8), and 1.6% type II (n = 2). Type IV was most commonly found in serotypes 16 (n = 15) and 3 (n = 13). In addition, 17.3% (n = 30) of the isolates were undetermined, mostly in serotype 31 (n = 7), and only 11.6% (20 isolates) were classified in the non-disease-associated groups, as shown in Table 2. Statistical analysis showed that there was no significant association between serotypes and types of disease-associated groups.

In some cases, pigs may be carriers of virulent isolates of *S. suis* without presenting clinical signs, but this bacterial species is a normal inhabitant of the upper respiratory tract, and most isolates are usually considered nonpathogenic or non-disease-associated. The results of the current study indicate that multiplex PCR failed to identify this group of clinically healthy pig isolates, and its use in routine diagnosis may lead to a high percentage of false-positive results. Indeed, the original study reported that 77% of pig isolates in the UK were associated with disease and 3% were non-disease-associated according to their origins [15]. However, Scherrer et al. (2020) obtained contradictory results, showing that 34.7% of diseased pig isolates from Switzerland were classified as disease-associated, whereas 65.3% were non-disease-associated using this multiplex PCR method [16]. Our results suggest that the pathotyping PCR method described originally in the UK [15] is probably not useful to clearly differentiate between disease-associated and non-disease-associated strains in clinically healthy pig isolates. It would be interesting to include isolates collected from diseased pigs in future studies to confirm this. It is highly important that multiplex PCR be validated using more diverse *S. suis* strains from different geographic areas and origins of isolation.

Furthermore, the disease-associated marker genes described by Wileman et al. (2019) should be reconsidered, and additional markers should be identified. Recent studies based on genomic analysis proposed new genetic markers that could distinguish between pathogenic and nonpathogenic pathotypes, including *ofs*, *srtF*, RNA-binding protein (*SSU_RS09525*), and two hypothetical proteins (*SSU_RS09155* and *SSU_RS03100*) [13, 14]. Marker genes *SSU_RS09155*, *SSU_RS09525*, and *SSU_RS03100* demonstrated strong associations with pathogenic *S. suis* strains (96%) in North America [14]. The *ofs*+/*srtF* + genotype was also present in 74% of the US pathogenic *S. suis* isolates [13]. These marker genes should be evaluated in further studies with different geographic strains. A previous study demonstrated that the CDS2157 gene, which belongs to the Tex protein family, was absent in all highly virulent ST1 isolates and was specific to the I/WV (intermediate/weakly virulent) group [19]. This gene may be a candidate for evaluation.

An effective pathotyping scheme for distinguishing the pathotypes of *S. suis* is needed. This could lead to the establishment of a public health surveillance program by promoting a focus on strains with virulence and pathogenic potential from either swine or humans. In particular, it has potential application for predicting pathogenic pathotype strains in pig farms or pig production companies. It also couples monitoring pathotypes of *S. suis* isolates from raw pork products for food safety purposes and a reduction in zoonotic transmission of this pathogen through improved surveillance programs because poor quality food safety controls for raw pork products at slaughterhouses and wet markets have been suggested as a source of infection in humans [3]. Therefore, pathotyping tools should be continuously developed and further evaluated.

## Conclusions

The multiplex PCR pathotyping method for *S. suis* strains described by Wileman and colleagues could differentiate isolates into four types of disease-associated strains. Some types of disease-associated groups were significantly related to some CCs, such as type I for CC1 and type IV for CC25 and CC104. However, this multiplex PCR method is not useful for differentiating non-disease-associated from disease-associated isolates in the case of Thai clinically healthy pig *S. suis* isolates, although it works well for human isolates.

## Electronic supplementary material

Below is the link to the electronic supplementary material.

Supplementary Material 1

## Data Availability

All data generated or analysed during this study are included here and are available from the corresponding author on reasonable request.

## References

[1] Goyette-Desjardins G, Auger JP, Xu J, Segura M, Gottschalk M (2014). Streptococcus suis, an important pig pathogen and emerging zoonotic agent-an update on the worldwide distribution based on serotyping and sequence typing. Emerg Microbes Infect.

[2] Kerdsin A, Segura M, Fittipaldi N, Gottschalk M (2022). Sociocultural factors influencing human *Streptococcus suis* disease in Southeast Asia. Foods.

[3] Segura M (2020). *Streptococcus suis* Research: Progress and Challenges. Pathogens.

[4] Silva LM, Baums CG, Rehm T, Wisselink HJ, Goethe R, Valentin-Weigand P (2006). Virulence-associated gene profiling of *Streptococcus suis* isolates by PCR. Vet Microbiol.

[5] Berthelot-Hérault F, Morvan H, Kéribin AM, Gottschalk M, Kobisch M (2000). Production of muraminidase-released protein (MRP), extracellular factor (EF) and suilysin by field isolates of *Streptococcus suis* capsular types 2, 1/2, 9, 7 and 3 isolated from swine in France. Vet Res.

[6] King SJ, Leigh JA, Heath PJ, Luque I, Tarradas C, Dowson CG, Whatmore AM (2002). Development of a multilocus sequence typing scheme for the pig pathogen *Streptococcus suis*: identification of virulent clones and potential capsular serotype exchange. J Clin Microbiol.

[7] Weinert LA, Chaudhuri RR, Wang J, Peters SE, Corander J, Jombart T, Baig A, Howell KJ, Vehkala M, Välimäki N, Harris D, Chieu TT, Van Vinh Chau N, Campbell J, Schultsz C, Parkhill J, Bentley SD, Langford PR, Rycroft AN, Wren BW, Farrar J, Baker S, Hoa NT, Holden MT, Tucker AW, Maskell DJ (2015). BRaDP1T Consortium. Genomic signatures of human and animal disease in the zoonotic pathogen *Streptococcus suis*. Nat Commun.

[8] Willemse N, van der Ark KCH, Stockhofe-Zurwieden N, Smith H, Picavet DI, van Solt-Smits C, Wisselink HJ, Schultsz C, de Greeff A (2019). Clonal expansion of a virulent *Streptococcus suis* serotype 9 lineage distinguishable from carriage subpopulations. Sci Rep.

[9] Dong X, Chao Y, Zhou Y, Zhou R, Zhang W, Fischetti VA, Wang X, Feng Y, Li J (2021). The global emergence of a novel *Streptococcus suis* clade associated with human infections. EMBO Mol Med.

[10] Chen C, Zhang W, Zheng H, Lan R, Wang H, Du P, Bai X, Ji S, Meng Q, Jin D, Liu K, Jing H, Ye C, Gao GF, Wang L, Gottschalk M, Xu J (2013). Minimum core genome sequence typing of bacterial pathogens: a unified approach for clinical and public health microbiology. J Clin Microbiol.

[11] Kerdsin A, Akeda Y, Hatrongjit R, Detchawna U, Sekizaki T, Hamada S, Gottschalk M, Oishi K (2014). *Streptococcus suis* serotyping by a new multiplex PCR. J Med Microbiol.

[12] Lacouture S, Okura M, Takamatsu D, Corsaut L, Gottschalk M (2020). Development of a mismatch amplification mutation assay to correctly serotype isolates of *Streptococcus suis* serotypes 1, 2, 1/2, and 14. J Vet Diagn Invest.

[13] Estrada AA, Gottschalk M, Rendahl A, Rossow S, Marshall-Lund L, Marthaler DG, Gebhart CJ (2021). Proposed virulence-associated genes of *Streptococcus suis* isolates from the United States serve as predictors of pathogenicity. Porcine Health Manag.

[14] Estrada AA, Gottschalk M, Gebhart CJ, Marthaler DG (2022). Comparative analysis of *Streptococcus suis* genomes identifies novel candidate virulence-associated genes in North American isolates. Vet Res.

[15] Wileman TM, Weinert LA, Howell KJ, Wang J, Peters SE, Williamson SM, Wells JM, Langford PR, Rycroft AN, Wren BW, Maskell DJ, Tucker AW (2019). Pathotyping the Zoonotic Pathogen *Streptococcus suis*: Novel genetic markers to differentiate Invasive Disease-Associated isolates from Non-Disease-Associated isolates from England and Wales. J Clin Microbiol.

[16] Scherrer S, Rosato G, Spoerry Serrano N, Stevens MJA, Rademacher F, Schrenzel J, Gottschalk M, Stephan R, Peterhans S (2020). Population structure, genetic diversity and pathotypes of *Streptococcus suis* isolated during the last 13 years from diseased pigs in Switzerland. Vet Res.

[17] Kerdsin A, Akeda Y, Takeuchi D, Dejsirilert S, Gottschalk M, Oishi K (2018). Genotypic diversity of *Streptococcus suis* strains isolated from humans in Thailand. Eur J Clin Microbiol Infect Dis.

[18] Kerdsin A, Takeuchi D, Nuangmek A, Akeda Y, Gottschalk M, Oishi K (2020). Genotypic comparison between *Streptococcus suis* isolated from pigs and humans in Thailand. Pathogens.

[19] Zheng H, Lan R, Zheng X, Cui Z, Liu Z, Bai X, Ji S, Gottschalk M, Xu J (2014). Comparative genomic hybridization identifies virulence differences in *Streptococcus suis*. PLoS ONE.

