# Supplementary figures and images for: Evaluation of pathotype marker genes in Streptococcus suis isolated from human and clinically healthy swine in Thailand
Source: BMC Microbiol. 2023 May 16;23:133. doi: 10.1186/s12866-023-02888-9 (PMC10186705; doi:10.1186/s12866-023-02888-9)

## Slide 1
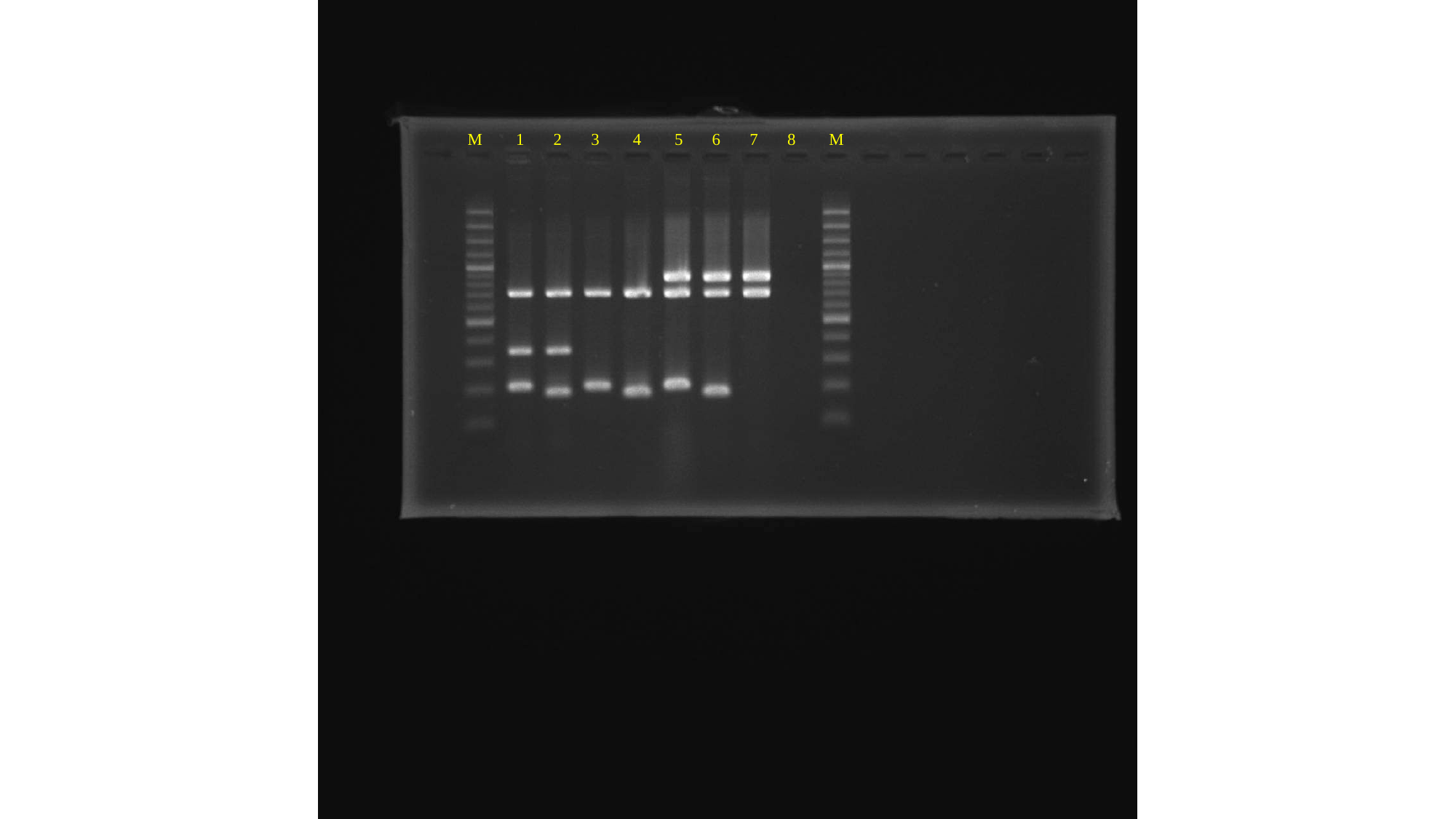

M 1 2 3 4 5 6 7 8 M

Supplement: Supplementary file 1 — Supplementary Material 1 [file 12866_2023_2888_MOESM1_ESM.pptx]
